# Laser Surface Modification of Aluminium Alloy AlMg9 with B_4_C Powder

**DOI:** 10.3390/ma13020402

**Published:** 2020-01-15

**Authors:** Marek Sroka, Ewa Jonda, Wojciech Pakieła

**Affiliations:** Department of Engineering Materials and Biomaterials, Silesian University of Technology, Konarskiego St. 18a, 44-100 Gliwice, Poland; ewa.jonda@polsl.pl (E.J.); wojciech.pakiela@polsl.pl (W.P.)

**Keywords:** aluminium alloy, laser surface alloying, microstructure, wear resistance, boron carbide

## Abstract

This paper presents the effects of laser treatment (fiber laser YLS-4000) on the microstructure and selected mechanical properties of the surface layer of AlMg (AlMg9) foundry alloy obtained by alloying with boron carbide (B_4_C). The correlation between laser alloying process parameters and selected properties of the formed layer was discussed. The studies were supported by microstructural analysis of the remelted zone (RZ), heat affected zone (HAZ), undissolved carbide particles, substrate material, and precipitates formed during rapid solidification. Metallographic investigations of the laser-treated layer were performed using optical microscopy and scanning electron microscopy (SEM). The elemental composition and a detailed analysis of chemical composition in micro-areas were carried out using energy dispersive X-ray spectroscopy (EDS). The remelting thickness, heat-affected zone (HAZ), and amount of base material in surface layers were determined. Microhardness tests were performed on transverse cross-sections of the remelted zone to obtain the hardness profiles in the base material (BM), remelted zone (RZ), and heat affected zone (HAZ). The hardness, roughness, and wear resistance measurements showed that the highest tribological properties of the obtained surface layer were achieved using 0.5 Bar protective gas (Ar) during alloying with B_4_C powder.

## 1. Introduction

The good ductility and lightweight nature of aluminium and its alloys have permitted their broad use in aerospace, automotive, and transportation industries. Aluminium also has high thermal and electrical conductivities, good machinability, and is easily recycled; however, this group of materials has relatively low mechanical and wear properties. Because of this, there is a need to improve the functional properties of these materials [1,2,3,4]. One of the primary methods to obtain materials with better mechanical properties is surface treatment technology. Laser surface alloying (LSA) is used for lightweight metals to improve their properties because the surface layer formed on the metal has different properties than the substrate material, for example, higher hardness, fatigue, and corrosion resistance; however, the surface is usually rougher than the original alloyed material [2]. LSA consists of enriching the surface layer with alloying elements, accompanied by structural changes. Usually, the alloying elements used in the laser treatment are metal alloys, superalloys, stellits, carbides, borides, and nitrides. LSA involves simultaneously melting and mixing the alloying material containing the alloyed additions with the treated material (base material) [5,6,7,8,9,10]. The laser beam fuses the base material, and a pool of remelted materials is created. Owing to convection and gravitation movements and the pressure of the laser beam, the materials are intensively mixed, and the properties of the formed layer depend on the microstructure, porosity, and chemical composition of the base material [11,12,13,14,15,16]. Boron carbide (B_4_C) has a high hardness (the third hardest material behind diamond and boron nitride), wear resistance, thermal conductivity, and melting temperature; however, its low strength (about 200–400 MPa) and low fracture toughness (2–3 MPa/m0.5), as well as poor sinterability, can significantly restrict its industrial applications [17,18].

Tian et al. [19] reported the effect of laser treatment parameters on the microstructure, microhardness, and wear resistance of pure titanium alloyed with B_4_C and Ti. The authors found that the depth of the remelted zone increased at lower scanning speeds. Additionally, the microhardness of the surface layer measured in cross-sections as a function of distance from the sample front decreased as the remelting zone depth decreased in a gradient. It has been reported that, compared with non-laser-treated surface materials, alloyed layers have excellent wear resistance, as well as a lower friction coefficient. Yilbas at al. [20] studied laser controlled melting of pre-prepared H12 hot work tool steel surface with B_4_C particles. The authors found that laser treatment reduced the friction coefficient of the surface layer, and the microhardness of the alloyed layer increased owing to the formation of nitrides, fine grains, and microstresses near B_4_C particles. In another case, Yilbas at al. [21] investigated the effect of laser surface modification treatment of aluminium bronze with B_4_C. The authors showed that the laser-treated surface was free of cracks, voids, and cavities, and the microhardness of the treated surface was significantly higher. Hlawka at al. [22] investigated chromium–molybdenum steel AISI 4135H surface hardened by laser melting with injected hard particles or by laser alloying using boron carbide or boron. They showed that laser melting of boron coatings produced very fine, uniformly-distributed microstructures in a remelting zone (RZ), and the surface had a good homogeneity without pores or cracks. The hardness was also higher than the substrate before laser treatment.

Tests were also conducted to cover the product with corrosion-resistant and harder phases (e.g., Al_2_O_3_) [23]. The effect of simultaneous melting and feeding of biphasic tungsten carbide WC/W_2_C particles into the molten pool on the structure and mechanical properties of ENAC-AlMg9 aluminium alloy was investigated [24]. For laser alloying, Cu, Mg, and Mn powders added to 98.6% aluminium using a CO_2_ laser were also used [25]. In contrast, Irek [26] presented the results of research on aluminium alloy AlSi7Cu4MgMn subjected to laser alloying using silicon carbide.

In spite of plenty of research, there is still not enough information about the microstructure and properties of the modification of surface layers ENAC-AlMg9 by laser alloying with the use of boron carbide (B_4_C). This manuscript is going to be an attempt to fill this gap as a current topic, from both a scientific and an application point of view.

## 2. Experimental Procedure

**Figure 1:**
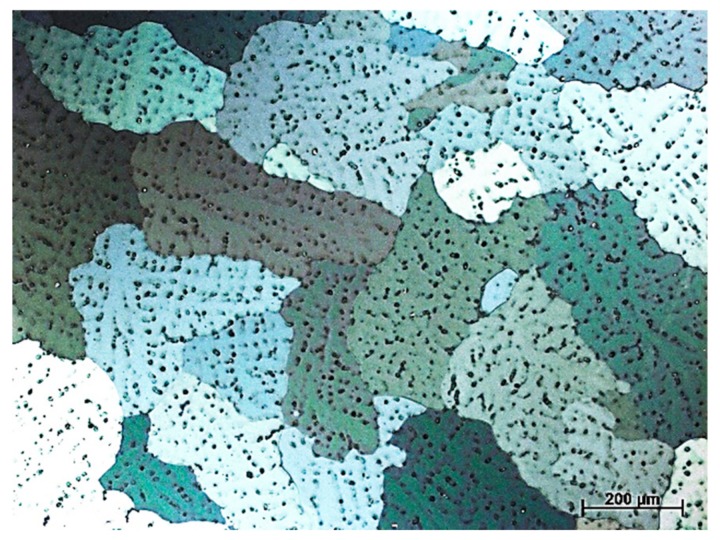
Microstructure of the AlMg9 aluminium alloy.

**Figure 2:**
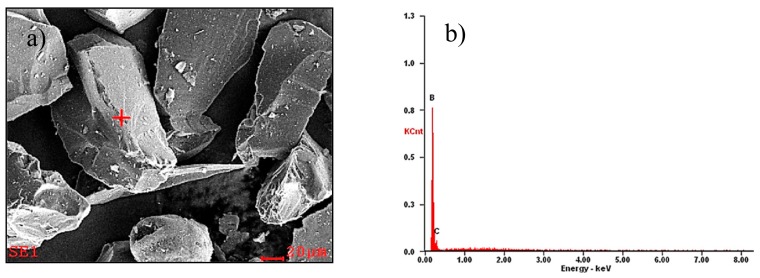
(**a**) Microstructure of the B_4_C powder (scanning electron microscopy (SEM)); (**b**) energy dispersive X-ray spectroscopy (EDS) analysis of the chemical composition of the B_4_C particle.

Investigations were carried out on test pieces from the casting aluminium alloy with magnesium ENAC-AlMg9 (Institute of Non-Ferrous Metals in Gliwice, Skawina, Poland). The chemical composition of the alloy is shown in Table 1, and the microstructure of the aluminium alloy used in the laser surface treatment is shown in Figure 1. The microstructure of the AlMg9 alloy in the casting state consists of the primary aluminium phase α-Al, which is the matrix of the alloy eutectic phase (Al + Mg_2_Si) and β-Al_8_Mg_5_. The development of the Al_8_Mg_5_ phase was observed at the boundaries of eutectic cells—between primary aluminium dendrites. The stoichiometric composition of the Mg_2_Si phase is 66.6 at% Mg and 33.4 at% Si [27]. To improve the properties of the surface layer, boron carbide (B_4_C) (Kamb Import-Export, Warsaw, Poland) powder was applied, which had the properties listed in Table 2. The average particle size of the powder was in the range of 63–106 µm. The carbide shapes determined with scanning electron microscopy (SEM) + energy dispersive X-ray spectroscopy (EDS) analysis, are shown in Figure 2a,b.

A fiber laser (FL) Ytterbium Laser System YLS-4000 (IPG Photonics Corporation, Oxford, MA, USA) was used for surface alloying, with a wavelength λ = 1070 nm, and a maximum laser beam power of 4000 W mounted on a six-axis robot REIS RV30-26 (Reis Robotics, Obernburg, Bavaria, Germany). The laser surface treatment was carried out under a shielding Ar gas to protect the molten weld pool. On the basis of preliminary experimental research regarding the impact of the shielding gas used on the depth and depth of the melted zone and the heat-affected zone for further studies, the best parameters were selected. The laser surface treatment was carried out using a constant alloying scanning rate of laser 0.2 m/min and laser beams power of 1.5 kW. The laser alloying parameters are shown in Table 3.

The topography of the alloyed surface was observed using a Zeiss stereomicroscope SteREO Discovery (Zeiss, Oberkochen, Germany) with magnification in the range of 10–100X. Specimens for metallographic observations were prepared by standard polishing techniques. Grinding at 25 N load successively on papers with grain gradation 120, 600, 1200, and 4000 and polishing with a colloidal suspension based on silicon oxide on a disc made of nephron rubber (MD-Chem). Electric etching was done in HBF_4_ acid (5% solution) for 20 s. Metallographic investigations done made using light microscopy with an Axio Observer and a Zeiss Supra 35 SEM (Zeiss, Oberkochen, Germany) using secondary electron and backscattered detectors. The chemical composition was analyzed by EDS. Hardness changes across the laser runs versus distance from the surface were investigated using the Vickers microhardness test method with a force of 500 gf. Hardness tests were performed along lines perpendicular to specimen surfaces, along the run face axis.

The resistance of the surface layers without laser treatment and after alloying with boron carbide was analyzed and compared using the “ball-on-plate” tribological test. As a counter-specimen, a 6 mm diameter ball of aluminium oxide Al_2_O_3_ was used. During the test, the friction coefficient between the investigated surface and ceramic counter was recorded. The test was performed at room temperature using the testing conditions in Table 4. The wear track dimensions after tests were measured by a Sutronic 25-Taylor Hobson profilometer (Taylor Hobson Ltd., Leicester, England), and the topography was analyzed using SEM to locate rifts and deformations on the surface layer owing to laser alloying with B_4_C carbide. The roughness of the investigated surface layer was also measured by a Sutronic 25-Taylor-Hobson profilometer.

## 3. Results and Discussion

**Figure 3:**
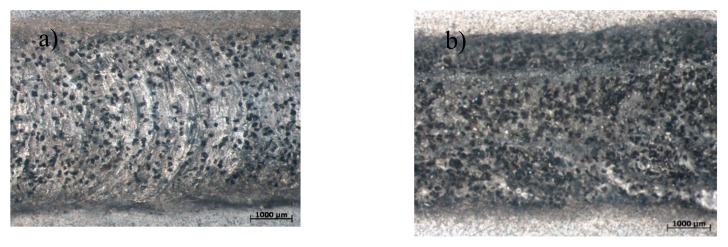
Topography of the layers obtained during laser alloying with B_4_C powder: (**a**) 0.4 Bar of protective gas; (**b**) 0.5 Bar of protective gas.

On the basis of the analysis performed here, the surface layer obtained owing to alloying an aluminium alloy with B_4_C powder was composed of three zones: a laser remelting zone (RZ), an enriched in boron carbide zone, a melted and rapidly solidified zone, and a heat-affected zone (HAZ). On the basis of preliminary experimental research regarding the impact of the shielding gas used on the depth and depth of the melted zone and the heat-affected zone for further studies, the best parameters were selected. The depth of the remelting layer obtained using 0.4 Bar of protective gas was about 1450 ± 18 µm, and the width was about 3833 ± 78 µm. When 0.5 Bar protective gas was used, the depth of the remelted layer was about 1642 ± 44 and the width was about 4132 ± 61 µm. The total surface layer thickness and width of both of the remelted zone and heat-affected zone grew when the pressure of the applied protective gas increased. Preliminary investigations of the alloyed aluminium ENAC–AlMg9 showed a clear effect of the laser treatment on the shape of the remelted material, the obtained run face showed characteristic flashes at the borders; however, no pores, cavities, or cracks were observed. The topography of the layers obtained by laser alloying with B_4_C powder are presented in Figure 3a,b.

**Figure 4:**
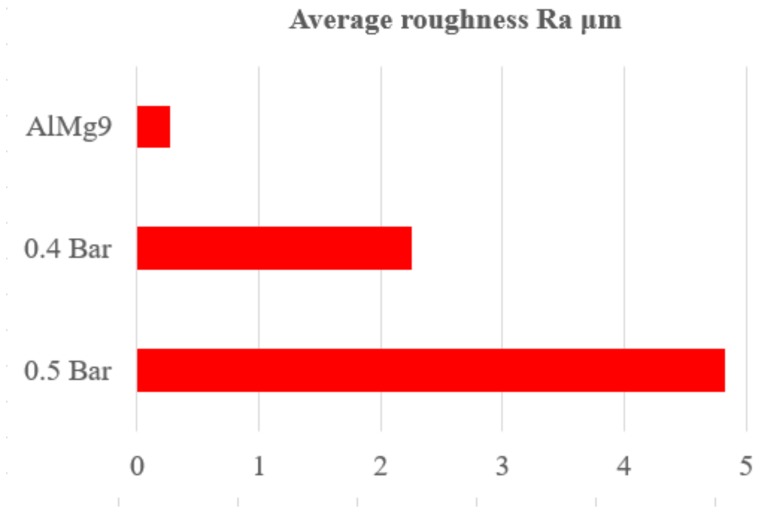
Effect of the laser alloying on surface layer roughness of the AlMg9 alloyed with B_4_C powder.

The roughness measurements show that during alloying ENAC–AlMg9 with B_4_C, the obtained run faces at both protective gas pressures had a higher roughness than the base material (BM) (average roughness of base material—*Ra* = 0.27 μm). The average roughness after alloying with 0.4 Bar of protective gas was 2.25 μm, and 4.82 μm with 0.5 Bar (Figure 4). The increase in roughness is closely related to the carbide amount introduced into the treated surface of the substrate material and the effect of shielding gas on the liquid metal, thus causing an increase in waviness. It should be emphasized that the roughness of the surfacing layer can be reduced to the desired value using grinding procedures and not as it is the case with physical vapour deposition (PVD) or chemical vapour depositinon (CVD) layers [29].

**Figure 5:**
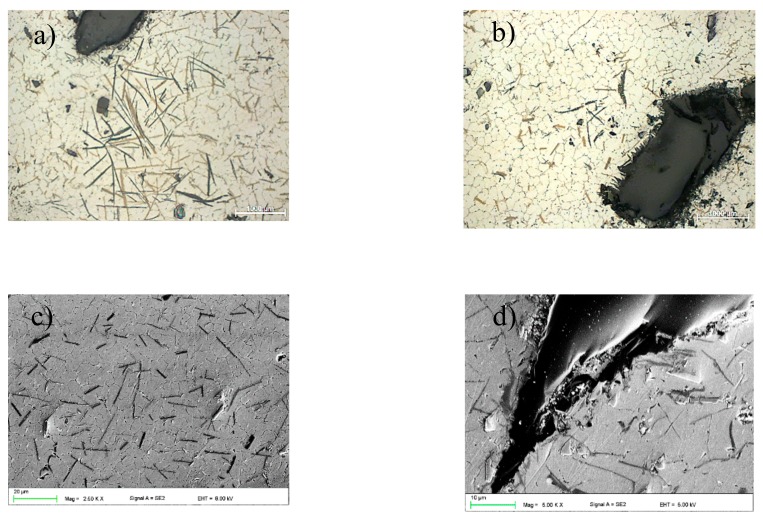
Microstructure of the layer obtained during the laser alloying with B_4_C powder (**a**,**c**) 0.4 Bar; (**b**,**d**) 0.5 Bar.

**Figure 6:**
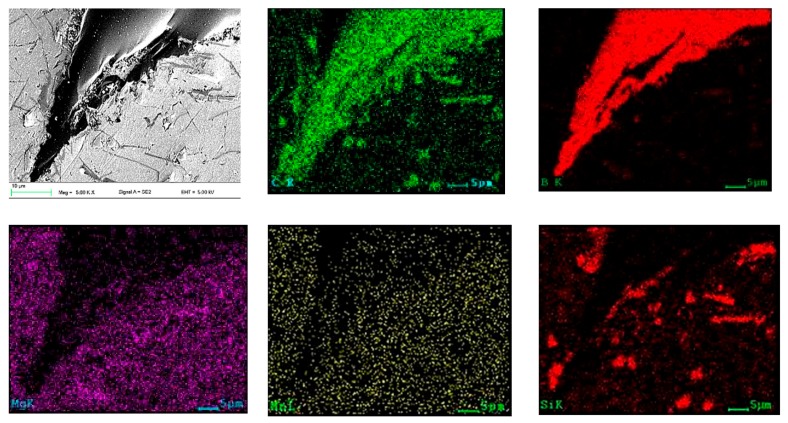
Elemental distribution maps of alloying elements in the analyzed area of the layer obtained during the laser alloying with B_4_C powder.

**Figure 7:**
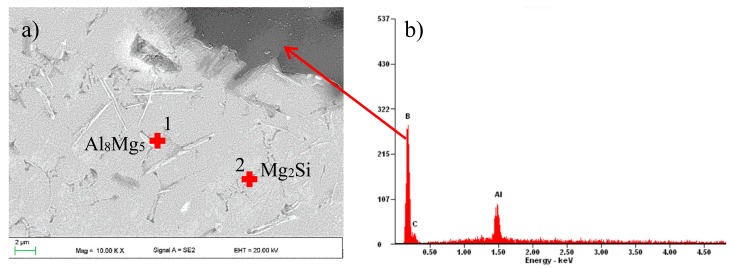
(**a**) Microstructure of the alloyed with B_4_C layer; (**b**) EDS analysis of the chemical composition of the analyzed point.

The microstructure of the solidified material after laser alloying contained areas with diverse morphologies owing to crystallization of the alloyed material (Figure 5a–d). When using 0.5 Bar protective gas, more presence of carbides as compared with 0.4 Bar Ar was observed. SEM observations showed that the applied B_4_C powder was evenly distributed in the remelted area (Figure 6), and the precipitates contained 90.83 wt% boron (Figure 7). In addition, around the disclosed carbides, zones of new separate phases were observed. EDS analysis showed that it consists of 46.70% at. coal and 53.30% at. silicon, which corresponds to the SiC phase (Figure 6 “C” and “Si”). Phases derived from substrate material rich in Mg and Al (in a ratio of 41.8/58.2% at) and Mg, Al, and Si (in a ratio of 39.2/37.9/22.9% at) were also disclosed, which correspond to the phases Al_8_Mg_5_ and Mg_2_Si.

AlMg9 without laser treatment showed a minimal friction factor, µ, of 0.72. The results showed that the coefficient of friction significantly decreased in the layers obtained during laser alloying with B_4_C. In the specimen alloyed with boron carbide, the average µ value was approximately 0.52 (for 0.4 Bar protective gas), whereas for 0.5 Bar protective gas, the average µ was approximately 0.42. On the basis of the investigation results, the friction coefficient was lower and fluctuated less in samples alloyed with B_4_C powder embedded in the surface layer compared with the native material. Initially, owing to the presence of partially-embedded boron carbide on the alloyed surface layer for all samples treated by a laser beam, the friction coefficient increased slightly. A decrease in the coefficient of friction was also observed for composites Al–B_4_C by Mazaheri et al. [30].

**Figure 8:**
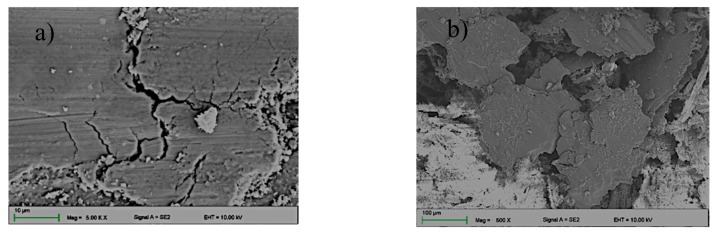
Wear trace (**a**,**b**) and the wear product (**b**) of the AlMg9 after the “ball-on-plate” wear test.

**Figure 9:**
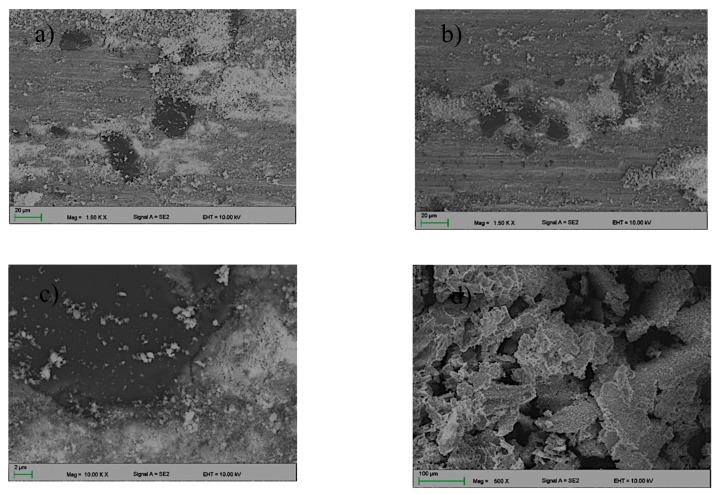
Wear track (**a**) 0.4 Bar, (**b**) 0.5 and the wear product, (**c**) 0.4 Bar, and (**d**) 0.5 of the sample alloyed with B_4_C powder after the “ball-on-plate” wear test.

**Figure 10:**
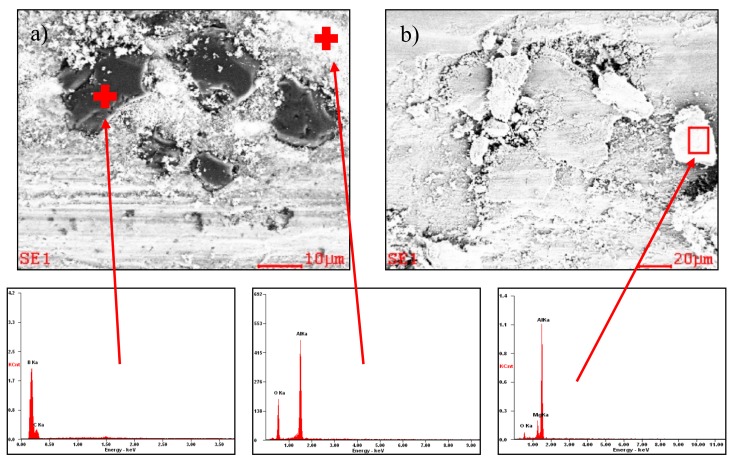
EDS microanalysis of the composite layer wear trace (**a**) and parental material (**b**).

The SEM topography observations of the substrate material abrasion showed various tribological wear mechanisms (Figure 8 and Figure 9). The most intense and dominant mechanism in all cases was abrasion wear (Figure 8a and Figure 9a,b). In addition, very intense delamination and plastic deformation were observed in the substrate (Figure 8a). The above mechanisms were also observed on the wear debris surface (Figure 8b). The analysis of the wear trace of the layer and wear debris (powder wear from the layer/wear product) showed no abrasion of large boron carbide particles debonded from the substrate, indicating good adhesion to aluminium. Wear debris observations showed significant differences in shape and size. Wear debris of the substrate occurred in the form of large flakes with sizes larger than 500 µm (Figure 8b), whereas the layers showed a mixture of fine dust and flakes smaller than 100 µm (Figure 9d), indicating much more even and stable wear. In addition, EDS microanalysis confirmed the occurrence of oxidation both on the surface of the wear trace of the layer and substrate material (Figure 10). Additionally, numerous agglomerations of fine oxidized wear debris smaller than 0.5 µm were observed on the surface of the wear trace of the composite layer. The wear trace dimensions after the “ball-on-plate” wear tests are shown in Table 5. Baradeswaran et al. [31] also showed a significant decrease in wear along with the increase in the participation of B_4_C carbide in alloy AA7075. Tribological wear of the composite with 10% carbide accounted for about 11% of the base material wear, which confirms the trend occurring in the case of laser alloying. In addition, the disappearance of plastic deformation was observed with the increase of B_4_C.

**Figure 11:**
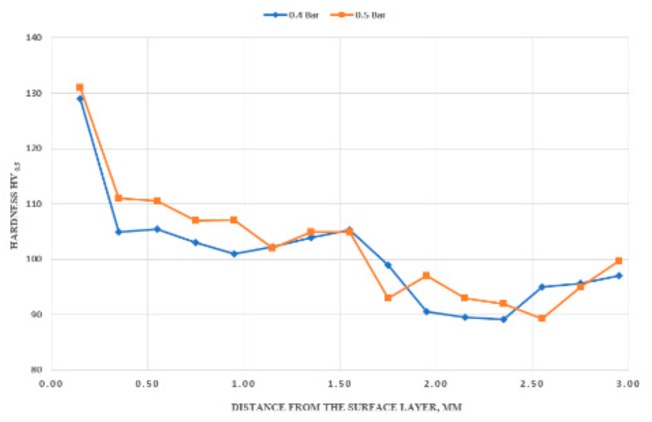
Profile of microhardness changes of the AlMg9 surface layer after laser alloying with B_4_C.

The hardness tests revealed that, when laser alloying with boron carbide, the resulting hardness was higher than the material before laser treatment. The measured microhardness along the depth of the cross-section of the solidified pool indicated a hardness increase to 128 HV_0.5_ (for 0.4 Bar protective gas) and 131 HV_0.5_ (for 0.5 Bar protective gas) only in carbide-containing areas at a depth of about 0.15 mm. The hardness drastically decreased in the entire heat-affected zone (HAZ) and along the border of the base material (BM). The hardness at a depth of 1.75 mm across the top surface layer ranged from 99–96 (for 0.4 Bar protective gas) and 93–99.9 (for 0.5 Bar protective gas) (Figure 11). The increase in hardness in the remelting area is caused by the presence of evenly distributed carbides and the fragmentation of precipitations coming from the substrate material. Baradeswaran et al. [31], introducing boron carbide particles into the 7057 alloy, obtained finally about 225 HB for the composite with 20% carbide. Boron carbide hardness is in the range of 2900–3900 kg/mm2, while the base material is slightly above 90 HV [32].

## 4. Conclusions

On the basis of the tests carried out on the AlMg9 alloy subjected to laser surface modification by rapid remelting and rapid solidification, the following conclusions can be made:

- The surface layer rich in alloying elements had a higher hardness than the substrate (128 HV_0.5_ for 0.4 Bar protective gas) and 131 HV_0.5_ (for 0.5 Bar protective gas).
- The abrasion resistance increased owing to an increase in the hardness of the surface layer. The obtained layers had friction coefficients of 0.52 (for 0.4 Bar protective gas) and 0.42 (for 0.5 Bar protective gas).
- An increase in tribological properties and a rougher surface (Ra = 4.82 µm) were found compared with the substrate before alloying (Ra = 0.27 µm).
- The hardness, roughness, and wear resistance measurements showed that the highest tribological properties of the obtained surface layer were achieved using 0.5 Bar protective gas (Ar) during alloying with B_4_C powder.

## Figures and Tables

**Table 1:** Chemical composition of aluminium alloy EN AC–AlMg9 (in wt. %).

Elements	Si	Mn	Zn	Mg	Al
AlMg9	1.32	0.50	0.20	9.24	REST

**Table 2:** Typical properties of B_4_C boron carbide powder [28].

Property	Value
Density, g/cm^3^	2.52
Melting point, °C	2445
Knoop hardness (100g), kg/mm^2^	2900–3580
Young’s modulus, GPa	450–470
Electrical conductivity (at 25 °C)	1.40

**Table 3:** Laser alloying parameters.

Parameter	Value
Laser beam power, kW	1.5
Protective gas, Bar	0.4
	0.5
The share of supplied powder, g/min	15
Laser beam scanning speed, m/min	0.2
Circle spot, mm	5
Wavelength λ, nm	1070

**Table 4:** Testing conditions of the “ball-on-plate” method.

Parameter	Value
Load, N	15
Linear speed, cm/s	5
Distance, m	50
Measuring distance, mm	6
Counter specimen	ball Al_2_O_3_

**Table 5:** The dimensions of the wear track of analyzed materials after the “ball-on-plate” wear test.

Substrate			Dimensions of the Wear Track		
			Volume, μm^2^	Width, mm	Depth, µm
AlMg9			193,603	1.99	55.5
AlMg9 +B_4_C	Protective gas, Bar	0.4	38,619	1.15	48.7
		0.5	26,313	1.2	43.2
